# The Impact of Search Engine Selection and Sorting Criteria on Vaccination Beliefs and Attitudes: Two Experiments Manipulating Google Output

**DOI:** 10.2196/jmir.2642

**Published:** 2014-04-02

**Authors:** Ahmed Allam, Peter Johannes Schulz, Kent Nakamoto

**Affiliations:** ^1^Institute of Communication and HealthFaculty of Communication SciencesUniversity of Lugano (Università della Svizzera italiana)LuganoSwitzerland; ^2^Marketing DepartmentPamplin College of BusinessVirginia TechBlacksburg, VAUnited States

**Keywords:** consumer health information, search engine, searching behavior, Internet, information storage and retrieval, online systems, public health informatics, vaccination, health communication

## Abstract

**Background:**

During the past 2 decades, the Internet has evolved to become a necessity in our daily lives. The selection and sorting algorithms of search engines exert tremendous influence over the global spread of information and other communication processes.

**Objective:**

This study is concerned with demonstrating the influence of selection and sorting/ranking criteria operating in search engines on users’ knowledge, beliefs, and attitudes of websites about vaccination. In particular, it is to compare the effects of search engines that deliver websites emphasizing on the pro side of vaccination with those focusing on the con side and with normal Google as a control group.

**Method:**

We conducted 2 online experiments using manipulated search engines. A pilot study was to verify the existence of dangerous health literacy in connection with searching and using health information on the Internet by exploring the effect of 2 manipulated search engines that yielded either pro or con vaccination sites only, with a group receiving normal Google as control. A pre-post test design was used; participants were American marketing students enrolled in a study-abroad program in Lugano, Switzerland. The second experiment manipulated the search engine by applying different ratios of con versus pro vaccination webpages displayed in the search results. Participants were recruited from Amazon’s Mechanical Turk platform where it was published as a human intelligence task (HIT).

**Results:**

Both experiments showed knowledge highest in the group offered only pro vaccination sites (*Z*=–2.088, *P*=.03; Kruskal-Wallis H test [H_5_]=11.30, *P*=.04). They acknowledged the importance/benefits (*Z*=–2.326, *P*=.02; H_5_=11.34, *P*=.04) and effectiveness (*Z*=–2.230, *P*=.03) of vaccination more, whereas groups offered antivaccination sites only showed increased concern about effects (*Z*=–2.582, *P*=.01; H_5_=16.88, *P*=.005) and harmful health outcomes (*Z*=–2.200, *P*=.02) of vaccination. Normal Google users perceived information quality to be positive despite a small effect on knowledge and a negative effect on their beliefs and attitudes toward vaccination and willingness to recommend the information (χ^2^_5_=14.1, *P*=.01). More exposure to antivaccination websites lowered participants’ knowledge (*J*=4783.5, *z*=−2.142, *P*=.03) increased their fear of side effects (*J*=6496, *z*=2.724, *P*=.006), and lowered their acknowledgment of benefits (*J*=4805, *z*=–2.067, *P*=.03).

**Conclusion:**

The selection and sorting/ranking criteria of search engines play a vital role in online health information seeking. Search engines delivering websites containing credible and evidence-based medical information impact positively Internet users seeking health information. Whereas sites retrieved by biased search engines create some opinion change in users. These effects are apparently independent of users’ site credibility and evaluation judgments. Users are affected beneficially or detrimentally but are unaware, suggesting they are not consciously perceptive of indicators that steer them toward the credible sources or away from the dangerous ones. In this sense, the online health information seeker is flying blind.

## Introduction

### Background

In 2012, more than 2 billion people worldwide used the Internet [1]. In the United States, 81% of adults reported Internet usage [2], whereas 73% of households in the United Kingdom had access to Internet [3]. One of the main purposes of Internet usage is seeking health information [2-6]. In fact, several studies reported that looking for medical information on the Internet was the first resort by individuals [2,4,5,7,8]. Despite the abundance of medical portals and health-related websites, many studies showed the main gateway for seeking health information was through search engines and, in particular, general search engines [2,9-11]. According to one study, 8 in 10 online health information seekers started with a search engine (Google, Bing, or Yahoo) [2].

In 2012, Google, the most popular search engine, processed as many as 1.2 trillion search queries in 146 different languages [12]. This number illustrates the importance of search engines for information seeking on the World Wide Web. A recent study [13] using “breast cancer” as a keyword found that results retrieved by 4 widely used search engines overlapped considerably, but each engine had a unique way of sorting/ranking (and thus emphasizing) various types of content. Because most searches produce many more results than anybody would be willing to read, people concentrate on the first results [10,14]. This gives the sorting/ranking algorithms of the search engines tremendous influence over the global spread of information and other communication processes. This study is concerned with demonstrating the influence of selection and sorting/ranking criteria operating in search engines on users’ knowledge, beliefs, and attitudes.

The promise of the Internet as a decision support system that makes available to patients not only a vast array of information, but also advice, product promotion, services, and even decision aids for health care and health maintenance [15] is seriously marred by the fact that the quality of health information on the Internet varies tremendously [16-24]. Many challenges arise from this variance, especially from the low-quality information content for people who might be literate enough to find, understand, and process such information and store it in their memories, but not literate enough to recognize it for what it is (false, irrelevant, or fraudulent). This is why, in the context of false or misleading health information, we speak of bad (or dangerous) health literacy [15], meaning the presence of the ability to understand medical information turned sour by the simultaneous absence of the ability to recognize it as false.

Quality criteria for health websites were developed in scholarly literature [17-19,24,25-31], but they cannot be expected to affect the everyday information-searching behavior of Internet users. At least 3 reasons exist for quality deficits making the Internet a potentially dangerous decision support system in matters of health. First, some Internet users might be incapable of telling high- from low-quality information. This problem was there before the Internet, but the sheer amount and diversity of health information available on the Web enlarges it considerably. Second, they might mistake the Internet as an authoritative source and not see the necessity of assessing the quality of the information. This is also not a new problem, and is likely increased by the specific nature of the Internet, including the easiness with which information can be retrieved. Third, users might mistake a search engine’s ranking of results as a quality ranking. This means they trust the search engine to provide the best websites and transfer this trust to the sites and the information they offer.

Quality deficits might result from knowledge deficits in the communicator because anybody can post health information on the Web. Quality deficits may also originate from material interests of communicators, such as businesses in the health sector that operate websites primarily as advertising for their products or services. Also, quality deficits may be caused by advocacy. As with commercial interests, strong advocacy in a controversial issue may mar the perception of facts relevant to the issue.

### Previous Studies

Early studies [16,20-24] discussed the challenges that arise from the variability of the quality of health information. Information overload, searching difficulties, and lack of organization, regulation, quality, and accurate information [23] are some of these challenges. Many initiatives and ideas were proposed to solve or reduce this problem. These initiatives included proposing guidelines or quality indicators for users [27-29,31], suggesting a seal of approval or code of conduct [20,27,28,30], promoting an eHealth code of ethics [32], evaluating website design features, and factors for boosting websites’ credibility [33-35]. Other studies suggested the use of rating systems, by giving the physicians and medical societies a role in applying filtering and labeling technologies [20].

The common methods used to assess the quality of the websites were to ask participants, patients, or physicians to rate a set of websites retrieved by multiple search engines [19,21,36-40]. Tools like DISCERN [41] and its brief version [42] were developed to assess patients’ written information with respect to treatment options; they were modified later for validating health information on the Internet. Many other studies have developed questionnaires that serve as measurements of the credibility and the quality of websites [34,35]. Google PageRank [43] may be a potential quality indicator after having medium correlation with ratings from DISCERN and the evidence-based quality of content [37]. Additionally, the readability level was also assessed for health-related websites [19,36], which showed that the majority of health-related websites required high school level or above [19,44]. This presented another challenge to understanding the online health material, especially for people with low literacy [44,45].

Not only was the quality of websites investigated, studies also evaluated the efficiency of search engines and the relevance of retrieved websites among multiple search engines. These studies proceeded by measuring the coverage and accuracy of the retrieved content [19] or by measuring the share of relevant content among the retrieved search results [38-40] from multiple search engines. Additional methods looked into the log files of search engines to understand what cancer-related queries people search for [8]. Another study combined transactional log analysis with a complementary pilot study to understand users’ online behavior and navigational trends at ClinicalTrials.gov [11]. In addition, keyword effectiveness indexing was applied and explored to estimate the ability of search engines to retrieve relevant results [46].

Other experiments observed participants in the laboratory during their online health information seeking [10,44,47-50]. Most of the studies started with a hypothetical scenario and asked the participants to perform a related search either for themselves or for others. The participants were audio/video recorded and their computer screen was also recorded. Additional log files were used for further analysis. Most of these studies [10,44,47-49] used a think-aloud protocol [51] in which participants spoke while they were searching. Some of the studies contained in-depth interviews [10,52,49] and/or focus groups [10,48].

Other studies looked at eHealth literacy of college students aiming at a career in a health profession, by measuring their research skills [50,53]. These studies used the Research Readiness Self-Assessment (RRSA) based on the Information Literacy Competency Standards for Higher Education. The RRSA measures proficiency in obtaining health information, evaluating the quality of health information, and understanding plagiarism [50].

To our knowledge, few or none of the previous studies manipulated a widely known search engine, such as Google, with the goal of systematically studying the effect of its sorting/ranking and selecting algorithm on users’ knowledge, beliefs, and attitudes toward a controversial health topic. By manipulating the search engine, we aimed at ascertaining the effects of using normal (unmanipulated) Google against a manipulated search on the beliefs and attitudes toward a particular health topic.

### Hypotheses

The subject for our study was the medical controversy around vaccination. Vaccination is one of the most important and influential medical discoveries, protecting and saving millions of lives [54-56]. In spite of its benefits, it has come into criticism and controversy instigated by antivaccination activists and organizations that have a strong online presence. The vaccination issue is one that plots medical evidence against lay skepticism or resistance [57]. The medical evidence considers the benefits of vaccination to clearly outweigh its risks. That means that the position against vaccination is only tenable if pertinent medical knowledge is disregarded or misrepresented. Therefore, in our view, website quality and position on the issue are intertwined and cannot be treated separately. We compared a search engine that delivered search results from high-quality provaccination websites with another that yielded lower-quality antivaccination websites.

Because websites and health interventions are known to increase knowledge [52,58-61] and high-quality sites contain more correct information on vaccination, we expect that users offered information (webpages) from high-quality websites by their search engine will gain more knowledge than users offered information from low-quality websites (hypothesis 1).

The effect of message tendency on recipient opinion may be the oldest theme in communication effects study. In light of this tradition, we expect that the higher the share of webpages (retrieved search results) from antivaccination websites offered by a search engine, the more critical users’ beliefs and attitudes on vaccination will become (hypothesis 2).

Favorable message and source assessment can be considered a prerequisite for communication effects [62-64]. There is much to say about source and message assessment than cannot be tested here (eg, how they are affected by pre-experimental attitude or how they change in the experiment). The concern here is with recognition of the website quality. We assume that people, even if they do not apply the whole catalog of scholarly quality criteria, can recognize the quality of websites (or the lack thereof). Consequently, users should assess high-quality provaccination websites more favorably than low-quality antivaccination sites (hypothesis 3).

## Methods

### Overview

We conducted 2 experiments to test our hypotheses. The independent variable in both experiments was the proportion of webpages (retrieved search results) from high-quality provaccination and lower-quality antivaccination websites, achieved by manipulating the search engine’s search space and sorting/ranking criteria. Dependent variables in both experiments were users’ knowledge, beliefs, and attitudes, as well as their assessment of the websites. Both studies were conducted as an experiment which set an information-seeking task to participants, directed them to customized search engines (without being aware of it), left their choice of search terms and their selection of sites (from among those offered by the search engines) uncontrolled, and measured the dependent variables immediately after.

### Experiment 1

#### Design

The first experiment was a pilot study to verify the existence of dangerous literacy in connection with searching and using health information on the Internet. Moreover, the goal was to explore the effect of 2 extreme manipulations of search engines that retrieved search results from either high-quality pro sites only or low-quality con sites only. A pre-post test design was employed to assess change, using the same questions and exact wording.

#### Experimental Conditions

Participants were allocated randomly to 3 experimental groups. Group 1 used normal Google, with its search coverage being the whole Web. Group 2 used Google configured to search for information from a set of websites certified by the Health on the Net (HON) code [30], which aims to provide health information of better quality and trustworthiness on the Web. The set additionally included websites from the World Health Organization (WHO), Public Health Agency of Canada, and governmental health agencies in the United States, such as the National Institutes of Health (NIH), Centers for Disease Control and Prevention (CDC), Food and Drug Administration (FDA), and other similar trustworthy credible websites. Group 3 used an engine configured to search for information from websites, blogs, and forums that discourage vaccination or were run by antivaccination activists and movements. Participants were unaware of the search engine manipulation and of which experimental group they belonged to.

#### Manipulating the Search Engine

The customization of the search engines was achieved by limiting their search coverage to different predefined sets of websites. This manipulation was realized by configuring the context and annotation files of Google custom search engine [65]. Both files were written in Extensible Markup Language (XML) in which the context file described the features and the settings of the search engine and the annotation file listed the set of websites the search engine covered in addition to weights and indicators instructing the search engine how information (webpages) retrieved from these sites would show or rank in the search results. Moreover, it allowed the possibility to widen the search space by including all webpages and documents found under the same domain. The first annotation file (for the search engine used by group 2) contained the HON-certified websites plus similar trustworthy and credible websites mentioned previously. The second annotation (for the search engine used by group 3) contained a set of websites that supported the con side of vaccination or discouraged people from vaccination. The set was assembled by running several regular searches on Google and other search engines for vaccination using negative keywords. Examples of the terms employed are vaccination and autism, side effects of vaccination, dangerous vaccines, antivaccination movements, antivaccination groups, bad vaccines, vaccine efficacy and skepticism, stop vaccination, etc. The sites yielded were read and informally classified as antivaccination or not. The search ended when the set of antivaccination websites contained 88 items. For the control group 1, we used normal Google (unmanipulated) to search the whole Web.

#### Procedure

The participants were informed that they were going to take part in an experiment about seeking online health information conducted by the University of Lugano in Switzerland. They were asked to inform themselves about vaccination and were given 10 minutes to do so. The search engines were embedded in a webpage that used the regular search layout provided by Google. We randomly distributed the instruction sheets of the experiment in front of the computers in the laboratory. Participants entered and chose a workstation as they wished. Each instruction contained a link to 1 of 3 webpages, which the participant was to open to begin the study. After answering a pretest questionnaire, participants were asked to search for information about vaccination for 10 minutes. Participants were free to search with any keyword they wanted and with as many as they wanted during the 10-minute time frame.

The retrieved search results were presented as a set of 10 search result pages; each page contained 10 results, adding up to a total of 100 websites/pages. A pager from 1 to 10 was displayed at the bottom of each page.

Strong emphasis was placed on the fact that this search engine was powered by Google and results were retrieved in a way so that participants were not alerted to any manipulation. After the search phase ended, they were redirected to answer a posttest questionnaire.

#### Recruitment and Participants

Marketing students from Virginia (USA) enrolled in a study-abroad program in Lugano, Switzerland, were asked to participate in the experiment. It ran in the computer laboratory of the University of Lugano. The sample of students was N=39 (group 1: n=12, group 2: n=14, group 3: n=13); 21% (8/39) were males and 79% (31/39) females.

#### Measures

Students had to fill out a pre-post questionnaire. The pretest questionnaire included the following measures used in the ensuing analyses:

1. Vaccination knowledge (or vaccination literacy): Battery of 14 true/false items combined to produce a knowledge index.
2. Attitude and beliefs toward vaccination: Set of 10 items that were presented as statements about vaccination, which participants responded to on a Likert scale (1=completely disagree to 7=completely agree). An additional 5 items measured the level of perceived side effects and benefits of vaccination for adults and children.
3. Sources and assessment of health information: A total of 7 items identified the participants’ main sources of health information and their trust in a set of predefined sources, measured on a Likert scale.
4. Sociodemographic items for gender, nationality, year of birth, level of education, type of work, and frequency of Internet usage. Two additional items were included if the participant had experience or worked in a medical environment and if the participant knew anyone who had a negative experience or side effect(s) from vaccines.

For the posttest questionnaire, students were asked to answer the following items:

1. Vaccination knowledge (or vaccination literacy) and attitude and belief toward vaccination: same as described previously.
2. Trust in retrieved information and consulted websites: A total of 7 items measuring summarily the credibility, satisfaction, trustworthiness, and relevance of the information retrieved and the websites visited.
3. Persuasion measure: A total of 5 categorical items measuring participants’ self-perceived persuasion by the sites visited.

#### Data Analysis

Wilcoxon signed rank test was used for testing significance within each group in a before/after experiment for the knowledge index and the different attitude and belief items. In addition, 1-way ANOVA and the Kruskal-Wallis H test were used to analyze if there was a difference in information quality assessment among the 3 groups.

### Experiment 2

#### Design

The second experiment was developed to leverage on the first and replicate it with a larger sample. It also increased the number of experimental conditions allowing for different ratios of pro and con sites in the mix provided by the search engine. Participants were again allocated randomly to one of the experimental conditions, and the procedure was similar to experiment 1; however, a posttest-only design was used.

#### Experimental Conditions

Experiment 2 compared 5 experimental groups and 1 control group who again used normal Google (group 1). The experimental groups differed in the ratio of con versus pro vaccination retrieved webpages offered by the customized search engine. Con and pro webpages were offered in the ratio of 0:10, 4:6, 6:4, 8:2, and 10:0 to groups 2 to 6, respectively. Group 2 (ratio 0:10) corresponds to group 2 in experiment 1, and group 6 (ratio 10:0) is similar to group 3 in experiment 1.

#### Manipulating the Search Engine

To manipulate the search engine for experiment 2, we started by writing context files and annotation files as in experiment 1, one pair describing the search engine restricted to a con vaccination set of websites and another pair to a pro vaccination set of websites (HON-certified and similar trustworthy credible sources reported in experiment 1). By using JavaScript and the Google custom search application programming interface (API) [65] without manipulating the ranking or the searching algorithm of Google, we programmatically controlled the search execution of both search engines and the display of the search results delivered from both [66]. The first search engine was called ConVaccineSearcher and the second ProVaccineSearcher. As the participant entered a search query in the provided search box, the 2 engines were launched with the same query and the results retrieved were displayed according to the participant’s experimental condition. For example, to achieve a ratio of 4:6, the first 4 results retrieved from ConVaccineSearcher were added on the top of the first 6 results retrieved from ProVaccineSearcher to build a search result page with 10 results.

#### Procedure

**Figure 1:**
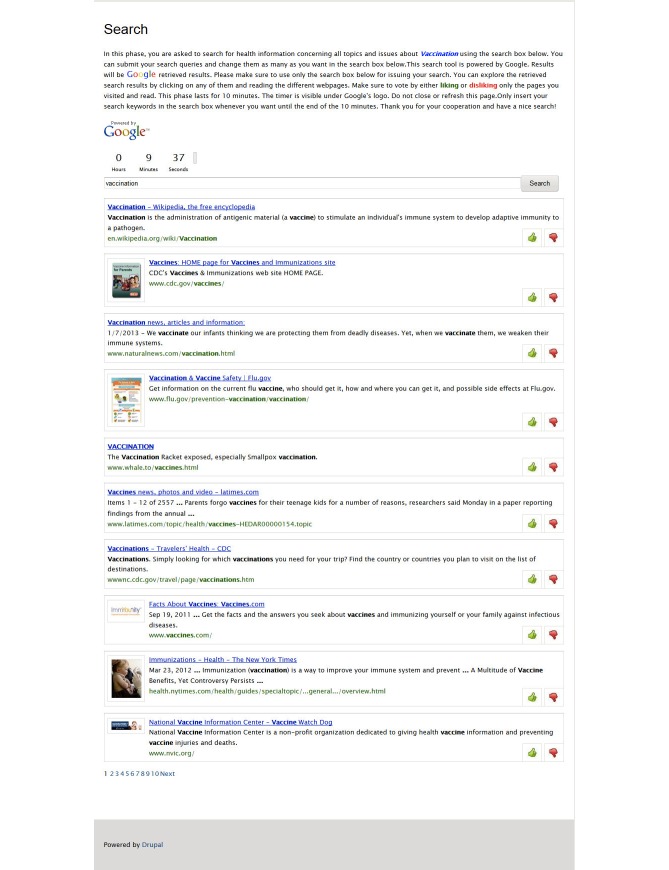
Screenshot of an unmanipulated Google search page.

Experiment 2 was presented as a website that proceeded in several steps similar to experiment 1. Figure 1 represents the search page presented to participants during the experiment. An additional like/dislike button was attached to every result (webpage). By clicking on these buttons, participants were able to rate the pages they read. As a visual indication for the rating action, the corresponding like/dislike button disappeared (Multimedia Appendix 1) once it was clicked. All the retrieved results (webpages), the like/dislike button, and the pager that represented a link to each of the 10 search result pages were attached to event handlers that updated the database using asynchronous JavaScript (AJAX), once they were clicked. This system was developed with the intention to record the behavior and the actions of each participant in a way that allowed constructing the timeline for each one.

After 10 minutes, an alert popped up informing the participants about the end of the search and redirecting them to the questionnaire. After completing the questionnaire, they were directed to a “thank you” page that displayed a generated code if the experiment was completed successfully. Multimedia Appendix 2 includes screenshots of these steps of the experiment.

Before the search and the questionnaire, participants were directed to a webpage (Multimedia Appendix 3) gateway that asked for the Amazon Mechanical Turk (MTurk) worker identification number before proceeding to the next step. MTurk is a crowdsourcing platform for requesting humans (MTurk workers) to work on executing specific tasks. In this way, we could track when they started the next phase and whether they passed through the experiment once or consecutively in one sitting. This ensured the validity of the flow of the experiment and allowed us to decide which participants followed the study instructions correctly.

#### Recruitment and Participants

For experiment 2, a Web platform using Drupal framework was developed. We designed a human intelligence task (HIT) for each of the groups that explained the experiment in general terms asking would-be participants to go to the Web platform we prepared to start. We used the same template and wording of the HIT for all groups. We published a HIT, renewed each week, on the MTurk platform for approximately 6 weeks (October 9 to November 19, 2012) that included an interruption because of technical difficulties. Each HIT linked to the previously mentioned Web platform that allocated the participants to the different manipulations. JavaScript syntax was embedded in the webpage and controlled the workflow of loading, choosing the custom search engine, and allocating the participants by randomly generating a number from 1 to 6 when the page loaded. Participants who were registered as Amazon MTurk workers and who passed the qualification requirements described subsequently were able to preview the HIT and apply for it if they wanted. At the end of each week, we analyzed the submissions and accepted people who followed the experiment as explained. The qualification requirements for the HIT were 1-Number of HITs approved greater than or equal to 5000. The 2-HIT approval rate for all requesters’ HITs was greater than 94%. The duration to complete the task was set to 30 minutes.

The sample size was N=197 (group 2: n=30, group 3: n=45, group 4: n=32, group 5: n=31, group 6: n=29, control group 1: n=30); 61.4% (121/197) were males and 38.6% (76/197) females. The nationalities of the participants were 0.5% (1/197) from England, 0.5% (1/197) from Hungary, 8.1% (16/197) from India, and 90.9% (179/197) from the United States. The participants’ level of education was 17.8% (35/197) with high school level, 81.2% (160/197) with college or university level, and 1.0% (2/197) with vocational training. The mean age of the participants was 37.32 years (SD 11.39). The minimum age was 20 years and the maximum was 69 years. Additional information about the age and the self-reported profession of the participants for each group is represented in Multimedia Appendix 4.

#### Measures

In experiment 2, participants also had to fill a questionnaire after the search. It was similar to the posttest questionnaire in experiment 1 with the addition of:

1. Persuasion measure: A total of 9 items measuring participants’ self-perceived persuasion by the sites visited on a Likert scale (versus the 5 categorical items in experiment 1).
2. Sociodemographic items that were presented in the pretest questionnaire of experiment 1.

#### Data Analysis

Knowledge scores were calculated for each participant and then ranked to apply the Kruskal-Wallis H test for testing for any significance among the experimental groups.

For the items measured on interval scales, we conducted factor analysis to find the latent variables [67]. Factor scores were calculated using Bartlett scores for each of the participants; Kruskal-Wallis H test, median test, and Jonckheere’s trend test were used for the analysis of scores between the experimental groups. Jonckheere’s test excluded the normal Google control group because the test is for a trend depending on decreasing and increasing shares of pro and con sites. The additional 2 items that indicated if the participants were persuaded by the information they read were analyzed using chi-square test. All analyses were conducted using SPSS 20 (IBM Corp, Armonk, NY, USA).

## Results

### Summary

All statistical analyses performed in this section were considered significant at *P*<.05. The Mann-Whitney *U* was used as a follow-up test for comparing different groups and a Bonferroni correction was applied; therefore, the significance level was changed to *P*<.01. Every time this occurs, it is clearly indicated. Moreover, we checked for the participants’ characteristics among the groups in both experiments to see if there was any bias in any of the groups. The sociodemographic measure including the 2 items mentioned previously was the criteria. Results were insignificant in both experiments, implying that the participants in the experimental groups were similar with no significant differences among them. These results are reported in Multimedia Appendix 5.

### Experiment 1

#### Knowledge

Knowledge questions from experiment 1 were calculated as scores and then ranked to apply the Wilcoxon signed rank test for checking any significance within each group in a before/after experiment. A significant increase in knowledge was observed in group 2 (*Z*=–2.088, *P*=.03), who were exposed to high-quality provaccination sites only. The other 2 groups did not show any significant increase in knowledge. An additional test was performed on the pretest knowledge scores of the 3 groups to check if there was any difference. The result was not significant (H_2_=4.02, *P*=.13)

#### Beliefs and Attitudes

Two of the 15 attitude measures showed a significant change within group 2 (only high-quality provaccination sites), but none in groups 1 and 3. Importance of vaccination in adults against influenza (*Z*=–2.326, *P*=.02) and the effectiveness of vaccination against swine flu (*Z*=–2.230, *P*=.03), group 3 (only antivaccination sites) showed an increase in concern about the side effects of vaccination for adults (*Z*=–2.582, *P*=.01) and believing that vaccinations cause more harm than good (*Z*=–2.200, *P*=.02). In the remaining 11 measures, no significant change was observed.

#### Assessment of Information

Using 1-way ANOVA and Kruskal-Wallis test showed no difference between the 3 groups for trust in the information found (*F*_2,36_=1.83, *P*=.17; χ^2^_2_=2.5, *P*=.28), satisfaction with the information found (*F*_2,36_=1.84, *P*=.17; χ^2^_2_=1.2, *P*=.54), assessment of its persuasiveness (*F*_2,36_=0.99, *P*=.38; χ^2^_2_=0.7, *P*=.68), information relevance (*F*_2,36_=2.97, *P*=.06; χ^2^_2_=5.4, *P*=.06), and trust in Google (*F*_2,36_=3.07, *P*=.06; χ^2^_2_=4.2, *P*=.12). This means none of the measures employed produced any significant differences between the experimental groups.

### Experiment 2

#### Knowledge

A knowledge index was computed for each participant based on the answers of 14 true-false questions in experiment 2. By using the Kolmogorov-Smirnov (*D*) and the Shapiro-Wilk tests, the distribution of the knowledge index (*D*_197_=.10, *P*<.001) was found to be significantly nonnormal. As a result, we opted for nonparametric tests. The Kruskal-Wallis H test showed that the knowledge index was significantly affected by the different exposition to con vs pro vaccination websites (H_5_=11.30, *P*=.04).

**Figure 2:**
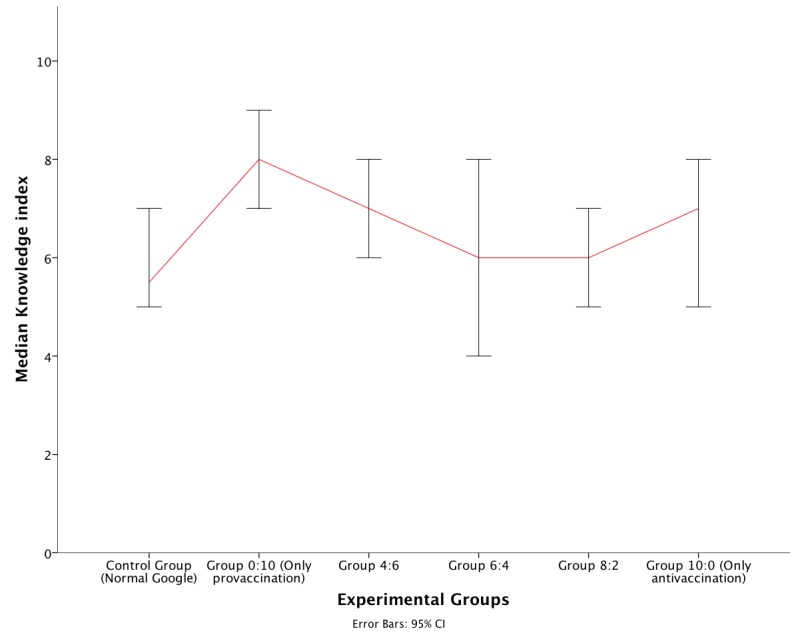
Median vaccination knowledge scores and 95% CI (error bars) by experimental group (experiment 2).

Mann-Whitney *U* tests were used to follow up this finding. A Bonferroni correction was applied and the effects were reported at .01 level of significance. The 4 comparisons between each of the groups 3 through 6 and normal Google (control group) did not show any significant result. Only group 2 showed a significantly higher level of knowledge versus the control group (*U*=241.5, *r*=–.4, *P*=.003). Figure 2 shows the medians for the experimental groups. It can be seen that as the share of search results belonging to antivaccination websites displayed increases, the median knowledge score decreases. In addition, the median of each group subjected to antivaccination websites is below the grand median.

Jonckheere’s trend test was applied to investigate whether the trend visible in Figure 2 was statistically significant. We hypothesized that the median of the knowledge index would decrease as the share of retrieved webpages from antivaccination websites in the search result pages increased. Jonckheere’s test revealed a significant trend in the data: the more participants were exposed to antivaccination websites, the lower the median of knowledge (*J*=4783.5, *z*=−2.142, *P*=.03).

In summary, the results from both experiments support our first hypothesis that users offered webpages from high-quality websites by their search engine will gain more knowledge than users offered webpages from low-quality websites.

#### Beliefs and Attitudes

**Figure 3:**
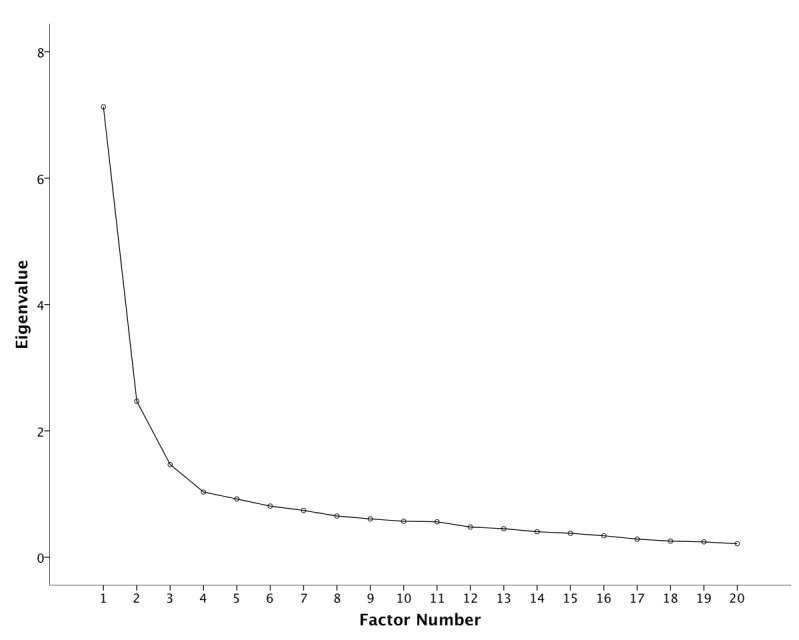
Scree plot for the factor analysis.

In the factor analyses of attitudinal measures, an additional step was performed to check for multicollinearity by evaluating the variance inflation factor (VIF), making sure the items under analysis maintained low levels of VIF (<3). The factor analysis ran on 21 items with oblique rotation (promax) using the maximum likelihood extraction method. The Kaiser-Meyer-Olkin (KMO) measure verified the sampling adequacy for the analysis (KMO=.894), and by looking at the diagonal of the anti-image correlation matrix, all KMO values for individual items were >.70, well above the acceptable limits. Bartlett’s test of sphericity (χ^2^_190_=1784.1, *P*<.001) indicated that correlations between items were sufficiently large for factor analysis. An initial analysis was run to obtain eigenvalues for each component in the data. Four eigenvalues were greater than Kaiser’s criterion of 1 and in combination explained 60% of the variance. We conducted a parallel analysis and looked into the scree plot in Figure 3, in which an inflection point was detected on the fourth eigenvalue. For this reason, we retained 3 factors for our final analysis. To assess the reliability of the subscales that emerged from factor analysis, we measured Cronbach alpha, which is the most common measure of scale reliability. Table 1 shows the pattern matrix of the factor analysis including each item’s loadings on the obtained 3 factors. The eigenvalues were 7.13, 2.46, and 1.46 and the percentage of variance explained by each from the total variance was 35.64%, 12.34%, and 7.33%, respectively. In addition, the Cronbach alpha for each subscale that comprised items that had greater than or equal 0.4 loadings was .88, .86, and .75 respectively. The items that form factor 1 suggest that it represents skepticism/fear of vaccination side effects, acknowledgment of vaccination benefits for factor 2, and information qualities for factor 3. Factors 1 and 2 were negatively correlated (–.67) whereas both were almost zero correlated with factor 3.

To compare the groups, factor scores were calculated using the Bartlett method. Applying the Kruskal-Wallis H test revealed that the level of fear for vaccination side effects was significantly affected by the different exposure to con versus pro vaccination websites (H_5_=16.88, *P*=.005).

**Figure 4:**
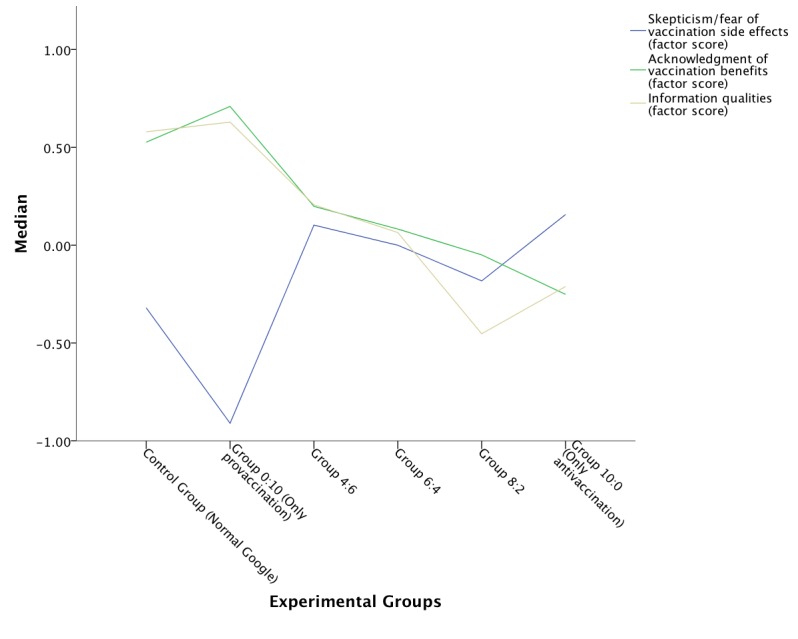
Median of the skepticism/fear of side effects, acknowledgment of benefits, and information quality factor scores by experimental group (experiment 2).

Mann-Whitney *U* tests were used to follow up this finding. A Bonferroni correction was applied and the effects were considered significant at .01 alpha level. The 5 comparisons between each of the groups 1, 3, 4, 5, and 6 and group 2 (offered only high-quality provaccination sites) showed significant results, as reported in Table 2. Figure 4 shows the median of factor 1 score by the experimental groups. The most important comparison was with the real scenario that included normal Google (control group). Normal Google and group 2 differed, but the other groups did not differ from the control group. Thus, normal Google offered websites that created as much fear of vaccination as any of the customized search engines.

**Table 1:** Factor analysis of attitude and website assessment measures in experiment 2.

Pattern matrix^b^	Factor loadings		
	1	2	3
When recommending vaccination, doctors do not pay enough attention to side effects	.807^a^	.093	.055
In your opinion, how serious are the side effects of vaccination on adults?	.746^a^	.016	–.027
Many vaccinations today do more harm than good	.705^a^	–.127	.035
Many vaccinations recommended today are not really necessary because the disease is more or less extinct	.674^a^	–.106	.069
In your opinion, how serious are the side effects of vaccination on kids?	.668^a^	–.117	–.007
People who vaccinate run a risk of getting the disease from the vaccination	.630^a^	–.055	.067
When I read in the websites about the efficacy of vaccination I felt worried	.621^a^	–.011	–.054
Vaccination often does not really protect against a disease	.583^a^	–.207	.108
Health authorities should put the necessity of vaccination programs to the test more often	.564^a^	.348	.104
In your opinion, should adults get vaccinated for influenza?	–.528^a^	.040	.219
In your opinion, should babies get vaccinated for Hepatitis B?	–.402^a^	.039	.085
In your point of view, how effective is vaccination against swine flu?	–.399^a^	.120	.205
Vaccination is one of the great medical breakthroughs affecting our lives	.122	.931^a^	–.053
If it weren’t for vaccination, many people today would have a shorter life span than they do	–.013	.794^a^	–.029
Sustaining and preserving current vaccination programs is a top priority of public health in our country	–.041	.686^a^	.118
People who opt against vaccination not only put themselves but also other people at risk	–.276	.568^a^	–.054
How much do you trust the information on vaccination you found in your search just now?	–.032	–.089	.779^a^
Was the information you found relevant?	.079	.094	.642^a^
I think that the information about vaccination I’ve read now from websites is comprehensible for me	–.111	–.092	.639^a^
How much do you trust Google to provide you with good information?	.034	.069	.582^a^

^a^Loading ≥0.4.

^b^Extraction method: maximum likelihood; rotation method: Promax with Kaiser normalization.

**Table 2:** Differences in user fears of vaccination between group 2 (only high-quality pro sites) and all others.

Group 2 vs:	Mann-Whitney *U*	Effect size *r*	*P*
Group 3	331	–.42	<.001
Group 4	252	–.40	.001
Group 5	280	–.34	.003
Group 6 (only con vaccination sites)	220	–.42	.001
Group 1 (normal Google)	282	–.32	.01

Regarding factor 2 representing the acknowledgment of vaccination benefits, Figure 4 displays the median factor score for different experimental groups. The Kruskal-Wallis H test showed a significant result (H_5_=11.34, *P*=.04). Using Mann-Whitney *U* tests with Bonferroni correction applied, significant results were reported at .01 alpha level. Only the 2 comparisons between groups 2, 3, and 6 showed significant results (*U*=444, *r*=–.28, *P*=.01 and *U*=273, *r*=–.31, *P*=.01, respectively).

To investigate whether there was a significant trend in the median of the attitude factor scores among different groups, Jonckheere’s trend test was applied. The groups were ordered 0:10, 4:6, 6:4, 8:2, 10:0, with the share of con vaccination websites rising step-by-step. Significant results are reported in Table 3.

Table 3 suggests that as the share of con vaccination websites displayed increased, the fear of vaccination side effects also increased, whereas the acknowledgment of vaccination benefits decreased. In other words as the con/pro ratio changed from 4:6 up to 10:0 (only con vaccination websites), participants became more and more fearful of vaccination and acknowledged less and less that it had benefits.

The presented results from both experiments support hypothesis 2 that the ratio of con and pro vaccination webpages offered by the search engine affects information seekers’ views on vaccination. The more con sites offered by the search engine, the more skeptical they became.

**Table 3:** Jonckheere’s trend test results for attitude factor scores.

Factor score	Jonckheere trend test score		*P*
	Observed, *J*	Standardized, *z*	
Factor 1: skepticism/fear of vaccination side effects	6496	2.724	.006
Factor 2: acknowledgment of vaccination benefits	4805	–2.067	.03

#### Assessment of Information

Factor 3 offered a first measure of information assessment, which found a significant result (H_5_=22.8, *P*<.001) in the Kruskal-Wallis H test. Comparisons that showed significant results after using Mann-Whitney *U* with Bonferroni correction at .01 alpha levels are reported in Table 4.

**Table 4:** Differences among groups in assessment of information quality.

Group comparison	Mann-Whitney *U*	Effect size *r*	*P*
Group 2 vs 5	216	–.45	<.001
Group 2 vs 6	231	–.40	.001
Group 1 vs 5	229	–.43	.001
Group 1 vs 6	242	–.38	.002

**Figure 5:**
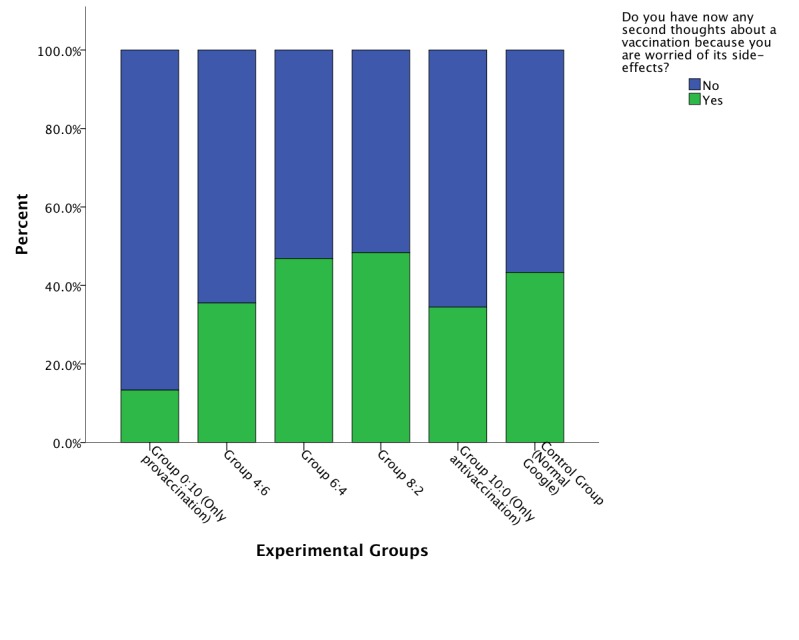
Bar graph of perceived persuasive effect of information retrieved by experimental group.

**Figure 6:**
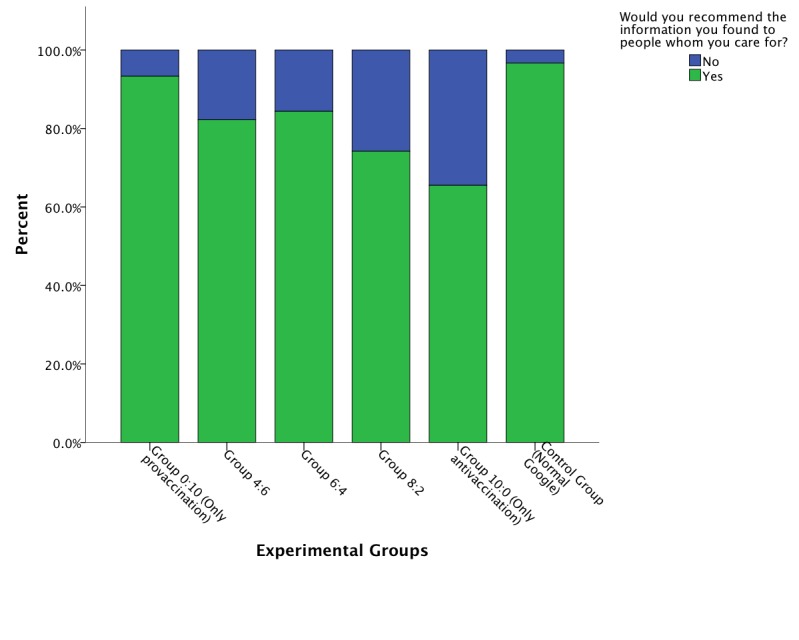
Bar graph of general assessment of information retrieved by experimental group.

Additionally, 2 items that are indicators of perceived information quality were analyzed using chi-square test. The items measured whether the participant perceived any opinion change toward vaccination and, as an indicator of general assessment of the information retrieved, would recommend the information found. The first item in Figure 5 resulted in χ^2^_5_=10.9, which corresponded to a value of *P*=.05 on the threshold of rejecting the null hypothesis. Participants in the group that did not receive lower-quality con sites, less often than the other groups attested to second thoughts after reading the information. Figure 6 shows the significant result (χ^2^_5_=14.11, *P*=.01) for the second item, indicating that when the share of the offered high-quality provaccination webpages decreased, the participants were less inclined to recommend to others the information they had retrieved. However, most in all groups recommended the information they found during the search phase.

The result from the first item implies that as the share of con vaccination websites increased, the doubt in vaccination efficacy increased. Moreover, the second item implied that health information seekers on the Web did recognize the quality of the sites they read to some extent, although most in all experimental groups recommended the information retrieved from the search engine.

We applied Jonckheere’s trend test for the information quality score as we did for the attitude scores. The test showed that as the share of displayed webpages belonging to low-quality con vaccination websites increased, the assessment of information quality decreased (*J*=4154, *z*=−3.911, *P*<.001)

Additional information regarding participants’ search terms is reported in Multimedia Appendix 6. The participants’ ratings of con and pro vaccination webpages is evident from the clicks on the like/dislike buttons. Table 5 displays the results. The webpages belonging to high-quality provaccination sites were liked more often and disliked less often than pages belonging to lower-quality antivaccination sites. The latter, however, were on average also liked. In fact, 83% of the judgments of the high-quality webpages and 64% of the judgments of low-quality pages were positive. The difference of approximately 20% was similar across the groups. The mix of sites offered did not affect the evaluation of the sites.

Table 5 shows that participants who were offered high shares of high-quality information looked at more pages than the groups offered high shares of low-quality information. Various interpretations can be considered. Lower-quality information (eg, may be more difficult to process) could make respondents stay longer on the respective sites. It could also be that high-quality information was a bit boring, which could make respondents move on faster.

**Table 5:** Overview of webpage choice and evaluation by using the like/dislike button (experiment 2).

Webpages evaluation		Experimental group (ratio con to pro sites)					Total N=167
		2 (0:10) n=30	3 (4:6) n=45	4 (6:4) n=32	5 (8:2) n=31	6 (10:0) n=29	
**Background**							
	Total unique webpages looked at, n	258	523	236	243	189	1449
	Total like/dislike clicks, n	451	1086	569	429	336	2871
	Webpages looked at per participant	8.6	11.6	7.4	7.8	6.5	8.7
	Like/dislike clicks per participant	15.0	24.1	17.8	13.8	11.6	17.1
	Like/dislike clicks per webpage looked at	1.7	2.1	2.4	1.8	1.8	2
**Higher-quality provaccination webpages**							
	Webpages looked at,^a^ n	258	252	68	42	—	620
	Like/dislike clicks, n	451	533	189	63	—	1236
	Like clicks, n (%)	341 (75.6)	465 (87.2)	165 (87.3)	49 (77.7)	—	1020 (82.5)
	Dislike clicks, n (%)	110 (24.3)	68 (12.7)	24 (12.6)	14 (22.2)	—	216 (17.4)
**Lower-quality antivaccination webpages**							
	Webpages looked at,^a^ n	—	271	168	201	189	829
	Like/dislike clicks, n	—	553	380	366	336	1635
	Like clicks, n (%)	—	379 (68.5)	231 (60.7)	223 (60.9)	227 (67.5)	1060 (64.8)
	Dislike clicks, n (%)	—	174 (31.4)	149 (39.2)	143 (39.0)	109 (32.4)	575 (35.1)
**Proportion of lower-quality antivaccination webpages**							
	Proportion of lower-quality webpages in experimental manipulation (%)	0	40	60	80	100	
	Proportion of lower-quality webpages looked at (%)	0	52	71	83	100	

^a^Most information not available for control group.

Groups offered a more evenly distributed mix of search result webpages from high- and low-quality sites tended to pass evaluation of the site more often. This could mean that the impression of 2 equally strong camps triggered site quality evaluations in users, whereas users offered a more uniform mix in either direction seemed less motivated to indicate how they thought of it. Across all groups who had a choice to select either type of sites, bias ran in favor of low-quality antivaccination sites, which were opened more frequently than their share in the manipulated search engine results would suggest. This could mean the con sites were, even just from looking at Google output, more interesting than the pro sites.

The results from experiment 2 support hypothesis 3 that webpages from high-quality sites were assessed more positively than webpages from lower-quality sites, which might be indicative of the ability to recognize high-quality medical websites. The hypothesis was not corroborated by experiment 1.

## Discussion

### Principal Findings

The study presents a new type of experiment that manipulates the selection and sorting criteria of a general search engine to study their influence on users’ knowledge and attitude toward health-related topics (vaccination in our case). The study retrieved results from 2 search spaces, one that was comprised of websites that were HON-certified or ran by credible trustworthy sources (eg, WHO, governmental health agencies) and supported the pro side of vaccination, and the other space was the websites skeptical of vaccination. The search engine algorithm for matching the queries with the website and all other parameters used in the algorithm to retrieve the relevant results and determine their rank/order were left intact.

The only group with an increase in knowledge in experiment 1 was the group offered only HON-certified and similar trustworthy webpages, and this group also differed from normal Google in experiment 2. This implies that knowledge gain by vaccination websites would be largest if websites critical of vaccination were filtered out. The result that participants scored higher in the knowledge index when they were less exposed to antivaccination websites supports assumptions that lay behind the initiatives promoting a health code of conduct [30], health-related seals of approval [16], and eHealth standards [31,32], and also the observation that websites with a high-quality rating using DISCERN or low scores on readability (grade levels) also contained health-related seals of approval [36]. As in Lorence and Greenberg [68], our study promotes the importance of quality proxies and encourages search engines to give higher rank to websites passed these quality criteria.

With regard to attitudes and beliefs about vaccination, the group offered only HON-certified and similar trustworthy credible webpages was the only group with positive change in experiment 1, and was the only group with lower levels of skepticism about vaccination in experiment 2, differing from both the normal Google group and all other experimental manipulations. In spite of a trend element showing more positive opinions as the proportion of provaccination Web pages grew, the results seem to indicate that attitude and belief change toward more support for vaccination programs depends on the absence of counter standpoints. Thus, the attitudes favored by websites offered by search engines translate into users’ views on issues. Search engines’ selection of websites according to bias, if it were to occur, can be expected to affect Internet users’ views.

As to assessment of the information received, there was no difference between the groups in experiment 1 indicating participants were not able to tell good from bad sites. Actually, there was a trend element in experiment 2 showing the sites were assessed better the higher the share of HON-certified and other similar trustworthy sites was. But the lower-quality sites were not assessed as bad; rather, they were assessed as less good than the others. In other words, quality assessments are not completely independent of actual quality, but health information seekers seem to be blind to the fact that there are misleading and dangerous information sources on the Web. People were shown to hold positive views toward seeking health information even if they had been unsuccessful in such endeavors before [49].

This study finds 3 elements that together constitute bad or dangerous health literacy. The first element is the presence of bad information, caused by our experimental manipulation in this study, but certainly prevalent in reality. The variability in the quality of the content on the Internet has been demonstrated in many studies from the early stages of Internet adoption [16,20-24]. The second element is communication effects, in our case suboptimal knowledge gain and transfer of skeptical views into people’s heads when they are presented with a large share of webpages from low-quality websites. Finally, a limited capacity for judging the informational value of the website was demonstrated and this is supposed to be the crucial condition that makes people misinform themselves on the Web. This capacity is not totally missing, but a weak sense of knowing what makes good sites does not exempt one from the influence of the bad ones.

Interestingly, results retrieved by normal Google were not as informative and beneficial to participants as the results retrieved by the manipulated search engine that retrieved only high-quality provaccination websites.The group offered provaccination websites only learned more than the normal Google group became less skeptical of vaccination; their support for vaccination was comparable as was their perceived information quality. Moreover, the intention to recommend the information found during the search was highest in the normal Google group. In fact, the factor that represented information quality was uncorrelated with the attitude scores. This suggests that effects are apparently independent of users’ manifest of site credibility and evaluation judgments. Another supporting observation is that sites of low quality were rated comparatively positively and this could explain why the quality assessment appears to be unrelated to the effects.

### Limitations

The study may have some limitations that are common to many online health information-seeking experiments. The search task is necessarily hypothetical and might turn out to be especially problematic if the participant is not interested or affected by the searching scenario. We chose, however, a medical topic that is considered more general and spans a wide range of people, especially all age groups. Vaccination concerns everybody and becomes more visible at the time of epidemics or the spread of infectious diseases, such as the H1N1 pandemic in 2009/2010. Therefore, we believe educating people and making sure they have good knowledge about vaccination and its benefits serves a good purpose in such scenarios. Moreover, Fox and Duggan [2] reported that half of online health information searches are conducted on the behalf of other individuals; this could justify the hypothetical scenario because searching for someone else might be similar to searching hypothetically.

The study exploited the design of an online system that took care of the experiment’s workflow and, more importantly, the seamless tracking of the participants’ actions during the entire search phase. The system recordings with the questionnaires’ responses formed the measurements that we received from the participants. Many studies, specifically observational ones, use verbal protocols such as the think-aloud protocol to capture the online health information-seeking process. As a result, we believe such methods would result in richer details and could focus on aspects that might be missed by the online systems or the questionnaires. Therefore, the study could benefit by integrating and using such protocols as part of the measures, which completes the whole image and provides a deeper insight in the experiments.

### Conclusions

By emulating a real-life scenario of a health-information search, this paper aimed to demonstrate suboptimal outcomes of such searches, not to attack search engines. Instead, we acknowledge the importance of the advances in the technology and the algorithms that are used by Google or any other widely used search engine. More importantly, our aim is to raise attention for the need of intelligent filters on top of these prominent engines that will help in redefining the search experience by providing different representation and evaluation of health information content on the Internet. Moreover, the study seeks to present new experiments that could be exploited further for future research especially in the area of search engine manipulation.

In conclusion, users are affected, be it beneficially or detrimentally, but the quality of the source of this effect largely escapes them. This suggests they are not consciously aware of indicators that steer them toward the promising sources or away from the dangerous ones. In this sense, the health information seeker on the Internet is flying blind.

## Multimedia Appendix 1

Retrieved search results; each attached a like/dislike button and webpages 1,2 and 5 are rated.

## Multimedia Appendix 2

Screenshots of the steps in the experiment after the search phase that includes pop up window indicating the end of the search phase, questionnaire phase and “Thank you” page displaying the automatic generated code.

## Multimedia Appendix 3

A gateway webpage that requests Mturk Worker ID before the search phase and the questionnaire phase.

## Multimedia Appendix 4

The age (in years) and self-reported profession of the participants in the six groups.

## Multimedia Appendix 5

Randomization check for Experiment 1 and 2. The experimental groups are compared to check if there is significant difference among them with respect the socio-demographic measure.

## Multimedia Appendix 6

Search terms used by participants during the search experiment. The size of the label is proportional to the frequency of occurrence of the keyword among all the groups.

## Abbreviations

AJAX: asynchronous JavaScript
API: application programming interface
HIT: human intelligence task
HON: Health on the Net
KMO: Kaiser-Meyer-Olkin
MTurk: Mechanical Turk
RRSA: Research Readiness Self-Assessment
VIF: variance inflation factor
XML: extensible markup language
