# Supplementary figures and images for: The Impact of Search Engine Selection and Sorting Criteria on Vaccination Beliefs and Attitudes: Two Experiments Manipulating Google Output
Source: J Med Internet Res. 2014 Apr 2;16(4):e100. doi: 10.2196/jmir.2642 (PMC4004139; doi:10.2196/jmir.2642)

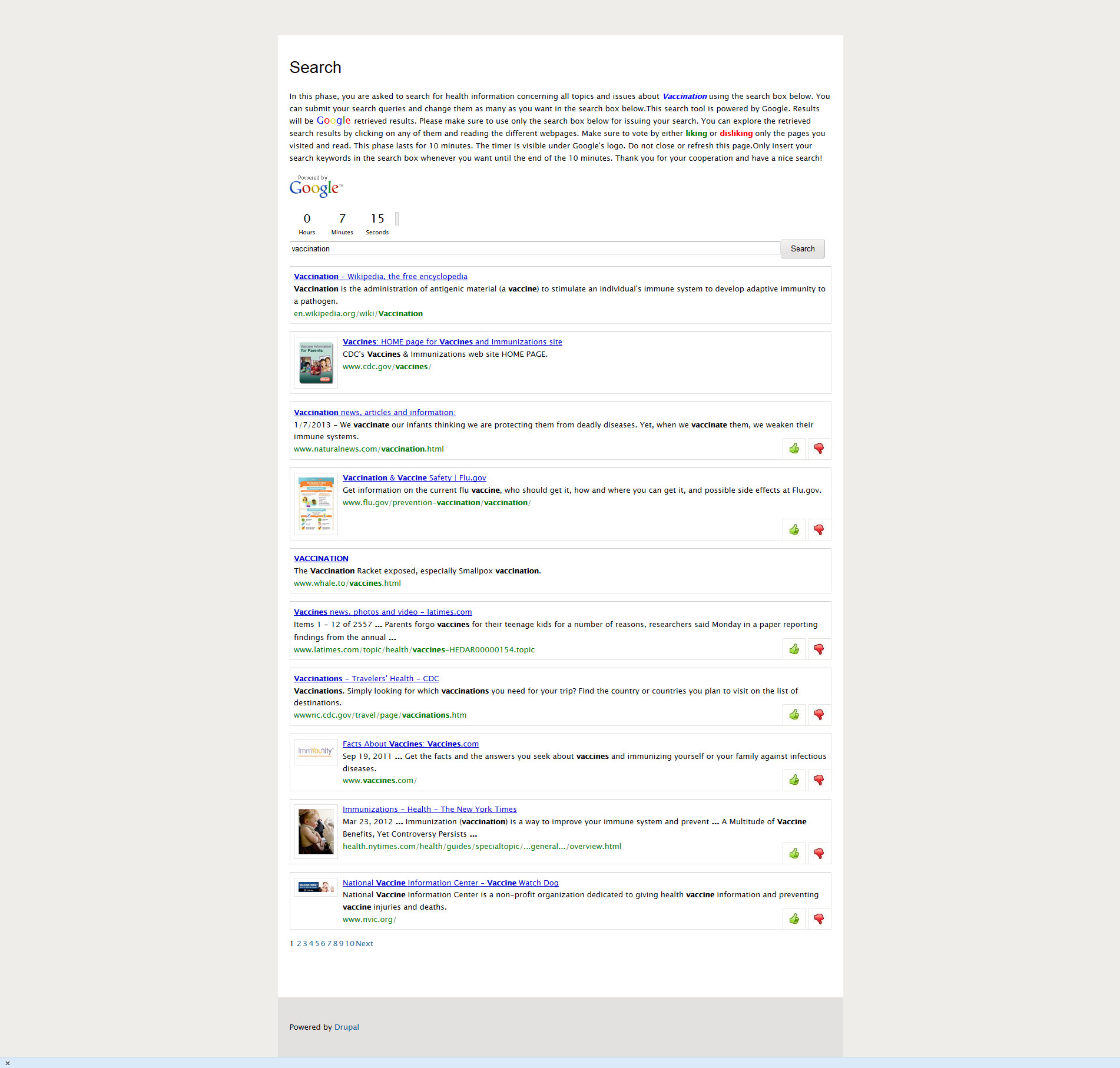

Supplement: Supplementary file 1 [file jmir_v16i4e100_app1.jpg]

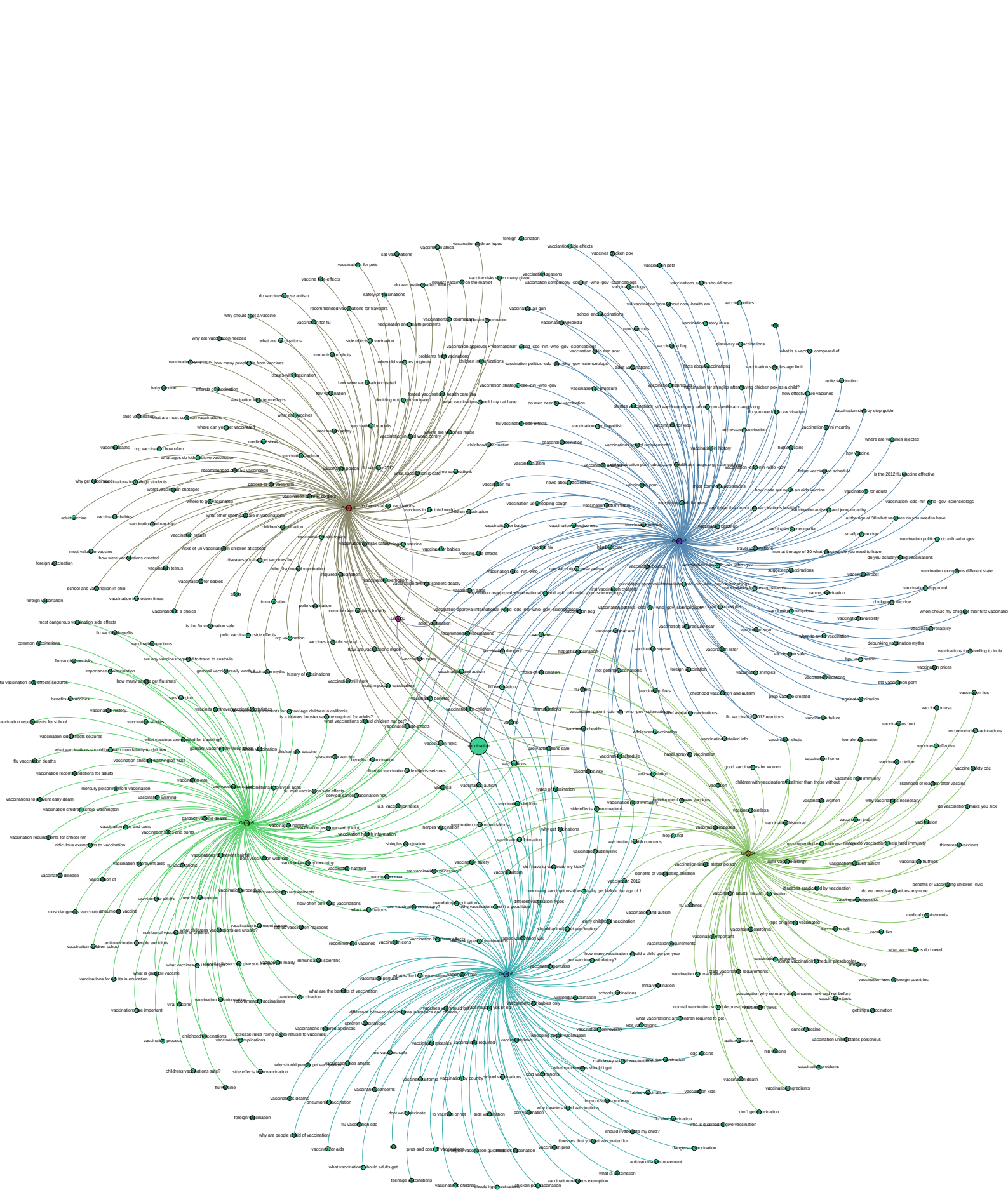

Supplement: Supplementary file 6 [file jmir_v16i4e100_app6.pdf]
