# Supplementary material for: The Impact of Search Engine Selection and Sorting Criteria on Vaccination Beliefs and Attitudes: Two Experiments Manipulating Google Output
Source: J Med Internet Res. 2014 Apr 2;16(4):e100. doi: 10.2196/jmir.2642 (PMC4004139; doi:10.2196/jmir.2642)
Supplement: Supplementary file 2 [file jmir_v16i4e100_app2.pdf]

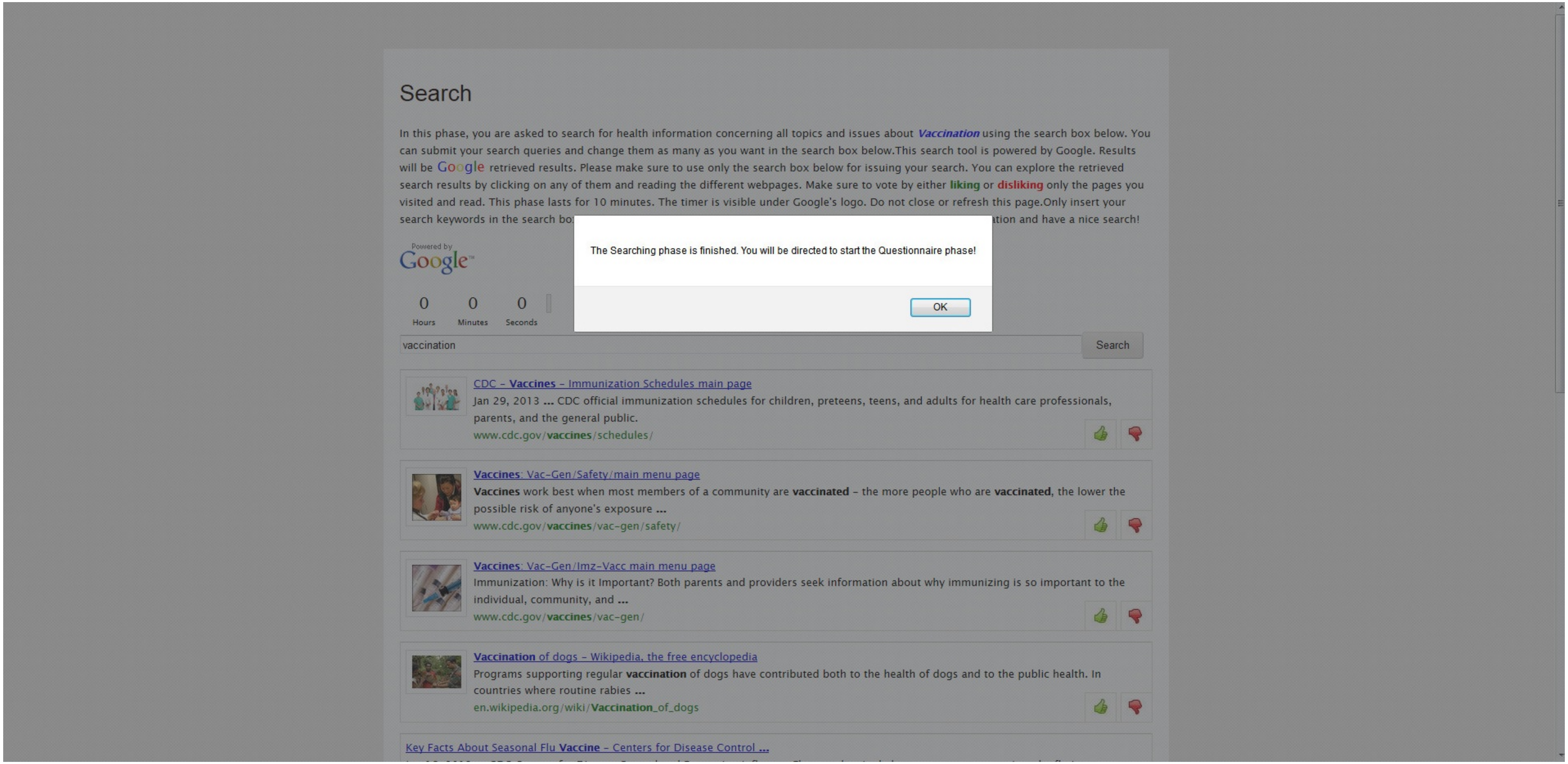
Search
In this phase, you are asked to search for health information concerning all topics and issues about Vaccination using the search box below. You can submit your search queries and change them as many as you want in the search box below.This search tool is powered by Google. Results will be Google retrieved results. Please make sure to use only the search box below for issuing your search. You can explore the retrieved search results by clicking on any of them and reading the different webpages. Make sure to vote by either liking or disliking only the pages you visited and read. This phase lasts for 10 minutes. The timer is visible under Google's logo. Do not close or refresh this page.Only insert your search keywords in the search bo
ation and have a nice search!
Powered by Google
0 Hours
0 Minutes
0 Seconds
The Searching phase is finished. You will be directed to start the Questionnaire phase!
OK
vaccination
Search
CDC - Vaccines - Immunization Schedules main page
Jan 29, 2013 ... CDC official immunization schedules for children, preteens, teens, and adults for health care professionals, parents, and the general public.
www.cdc.gov/vaccines/schedules/
Vaccines: Vac-Gen/Safety/main menu page
Vaccines work best when most members of a community are vaccinated - the more people who are vaccinated, the lower the possible risk of anyone's exposure ...
www.cdc.gov/vaccines/vac-gen/safety/
Vaccines: Vac-Gen/Imz-Vacc main menu page
Immunization: Why is it Important? Both parents and providers seek information about why immunizing is so important to the individual, community, and ...
www.cdc.gov/vaccines/vac-gen/
Vaccination of dogs - Wikipedia, the free encyclopedia
Programs supporting regular vaccination of dogs have contributed both to the health of dogs and to the public health. In countries where routine rabies ...
en.wikipedia.org/wiki/Vaccination_of_dogs
Key Facts About Seasonal Flu Vaccine - Centers for Disease Control ...

## Survey

Well done! You sucessfully completed the Search phase and now it is time for the Questionnaire phase. Please make sure to concentrate and read well the questions presented to you. You should only respond to the questions using your own information without the help of any other mean. Thank you again for participating in this study.

Next >>

Exit and clear survey

# Thank you for your participation

The code that you need to copy and submit to the Hit on Mturk page is: 414415123

Powered by Drupal
