# Supplementary material for: The Impact of Search Engine Selection and Sorting Criteria on Vaccination Beliefs and Attitudes: Two Experiments Manipulating Google Output
Source: J Med Internet Res. 2014 Apr 2;16(4):e100. doi: 10.2196/jmir.2642 (PMC4004139; doi:10.2196/jmir.2642)
Supplement: Supplementary file 3 [file jmir_v16i4e100_app3.pdf]

Fill in your Mturk Worker ID

To start the Searching phase, please fill in your Mturk worker ID in the form below.

Continue

Powered by Drupal

Fill in your Mturk Worker ID

To start the Questionnaire phase, please fill in your Mturk work ID in the form below.

Continue

Powered by Drupal
