# Supplementary material for: The Impact of Search Engine Selection and Sorting Criteria on Vaccination Beliefs and Attitudes: Two Experiments Manipulating Google Output
Source: J Med Internet Res. 2014 Apr 2;16(4):e100. doi: 10.2196/jmir.2642 (PMC4004139; doi:10.2196/jmir.2642)
Supplement: Supplementary file 4 [file jmir_v16i4e100_app4.pdf]

Descriptives

	Experimental Groups			Statistic
Year of birth	Group 0:10 (Only provaccination)	Mean		36.33
		95% Confidence Interval for Mean	Lower Bound	32.06
			Upper Bound	40.61
		5% Trimmed Mean		35.91
		Median		33.00
		Std. Deviation		11.454
		Minimum		20
		Maximum		61
	Group 4:6	Mean		36.22
		95% Confidence Interval for Mean	Lower Bound	32.81
			Upper Bound	39.63
		5% Trimmed Mean		35.54
		Median		32.00
		Std. Deviation		11.356
		Minimum		22
		Maximum		69
	Group 6:4	Mean		38.56
		95% Confidence Interval for Mean	Lower Bound	34.23
			Upper Bound	42.89
		5% Trimmed Mean		38.35
		Median		36.50
		Std. Deviation		12.013
		Minimum		20
		Maximum		61
	Group 8:2	Mean		37.87
		95% Confidence Interval for Mean	Lower Bound	34.29
			Upper Bound	41.45
		5% Trimmed Mean		37.62
		Median		37.00
		Std. Deviation		9.766
		Minimum		22
		Maximum		59
	Group 10:0 (Only antivaccination)	Mean		39.69
		95% Confidence Interval for Mean	Lower Bound	34.39
			Upper Bound	44.99
		5% Trimmed Mean		39.25
		Median		35.00
		Std. Deviation		13.939
		Minimum		21
		Maximum		66
	Control Group (Normal Google)	Mean		35.77
		95% Confidence Interval for Mean	Lower Bound	32.09
			Upper Bound	39.44

	5% Trimmed Mean	35.52
	Median	35.50
	Std. Deviation	9.849
	Minimum	21
	Maximum	56

Experimental Group	Self-reported Profession
**Group 0:10 (only provaccination)**	Manager - Retail Product Development
	Web Designer
	student
	Information Technology Consultant/Contractor
	Furniture restorer
	Artist
	Homemaker
	Sales Associate
	self-employed
	transcriptionist
	retail sales
	Entry level Scientist
	Unemployed
	homemaker
	Biomedical Engineer
	IT Manager, temporarily unemployed
	Teacher (Special Education)
	civil engineer
	Media Coordinator
	unemployed
	Insurance Sales
	Mathematician
	Student
	Freelancer
	IT
	Electrician assistant
	WAHM
	unemployed
	retired
	Software developer
**Group 4:6**	dog walker
	Independent Contractor
	marketing associate
	Hotel Management
	LMT
	Business Management
	clerical
	HOMEMAKER
	Accounts Manager
	retired
	sr executive in finance section
	unemployed

	Food Service/Delivery
	web designer
	data entry
	designer
	Legal Process
	Consultant
	IT (Hardware)
	Associate
	Technical
	software engineer
	Work at home
	p.c technician
	homemaker
	Software Engineer
	STUDENT
	SENIOR MANAGER
	cook
	HR manager
	Assistant Manager - Admn
	service
	embryologist
	nursing
	Tax Accountant
	Housewife
	Homemaker
	Field Engineer
	Teacher
	Unemployed
	Manager
	none
	Commercial Sales Manager
	homemaker
	freelance writer
**Group 6:4**	Aviation
	Day Trader
	Project writer
	retail management
	Image Editor
	Independent Contractor
	clerk
	Unemployed
	office manager
	Student

	sales rep
	Freelance writer & editor
	Teacher
	office clerk
	Administrative Assistant
	tutor
	Contractor
	Retail
	Magazine columnist
	manager
	Office Manager
	Sales
	Entertainer
	teacher
	homemaker
	Freelancer
	Meat Clerk
	Child Care Worker/self-employed
	unemployed
	retail sales
	analyst
	Student
**Group 8:2**	Farmer
	accountant
	Homemaker/Mom
	Lawyer
	Computer Programmer
	In home services provider
	Student
	homemaker
	Social Services
	process analyst
	bookkeeper
	X Ray Technician
	Analyst
	Licensed marriage and family therapist and writer
	transcriptionist
	mturker
	Manager
	Administrative Support Staff
	Administrative Assistant
	Admin Asst
	Barista

	Administrative assistant
	Customer Service Representative
	Self Employed Microtaskworker
	Heavy equipment operator
	HVAC Technician
	Customer support
	office worker
	Auto Mechanic
	Teaching assistant
	Homemaker
**Group 10:0 (only antivaccination)**	unemployed
	home health aide
	unemployed
	Librarian
	data reseacher
	homemaker
	Advice Worker
	salesman
	park maintenance
	Sales manager
	tutor
	Retail
	Admin. Assistant
	Customer Service Rep
	teaching
	media
	RADIO DJ
	journalist
	Lab Manager
	Legal secretary
	none
	self-employed
	unemployed
	Retired
	Student
	Automotive Mechanic
	Construction
	Teacher
	Inteligence Analyst
**Normal Google (Control Group)**	day trader
	Customer Service Representative
	Manager
	Surgical Technoligist

	Substitute Teacher/Tutor
	artist
	Self employed
	Media
	Television Broadcast
	Student
	Customer Service Agent
	Marketing
	Biologist
	student
	house keeping
	insurance
	student
	Medical Researcher
	Field Manager
	unemployed
	stay at home mom
	Financial Analyst
	Student/student librarian
	Homemaker\\Turker
	Tupperware Consultant
	School Counselor
	retail clerk
	IT Manager
	Operation Support (Law Firm)
	Freelance writer
