# Supplementary material for: The Impact of Search Engine Selection and Sorting Criteria on Vaccination Beliefs and Attitudes: Two Experiments Manipulating Google Output
Source: J Med Internet Res. 2014 Apr 2;16(4):e100. doi: 10.2196/jmir.2642 (PMC4004139; doi:10.2196/jmir.2642)
Supplement: Supplementary file 5 [file jmir_v16i4e100_app5.pdf]

Experiment 1

			Experimental Group			Total
			Control Group (Normal Google)	Group 0:10 (Only provaccination)	Group 10:0 (Only antivaccination)	
Did you ever know someone who had a bad experience from vaccines?	Yes	Count	1	3	2	6
		Expected Count	1.8	2.2	2.0	6.0
	No	Count	11	11	11	33
		Expected Count	10.2	11.8	11.0	33.0
Total		Count	12	14	13	39
		Expected Count	12.0	14.0	13.0	39.0

Chi-Square Tests

	Value	df	Asymp. Sig. (2-sided)	Exact Sig. (2-sided)
Pearson Chi-Square	.851[a]	2	.653	.856
Fisher's Exact Test	.895			.856
N of Valid Cases	39			

a. 3 cells (50.0%) have expected count less than 5. The minimum expected count is 1.85.

			Experimental Group			Total
			Control Group (Normal Google)	Group 0:10 (Only provaccination)	Group 10:0 (Only antivaccination)	
Have you ever had training for a job in the medical field or worked in a medical environment?	Yes	Count	0	3	1	4
		Expected Count	1.2	1.4	1.3	4.0
	No	Count	12	11	12	35
		Expected Count	10.8	12.6	11.7	35.0
Total		Count	12	14	13	39
		Expected Count	12.0	14.0	13.0	39.0

Chi-Square Tests

	Value	df	Asymp. Sig. (2-sided)	Exact Sig. (2-sided)
Pearson Chi-Square	3.363[a]	2	.186	.300
Fisher's Exact Test	2.781			.300
N of Valid Cases	39			

a. 3 cells (50.0%) have expected count less than 5. The minimum expected count is 1.23.

			Experimental Group			Total
			Control Group (Normal Google)	Group 0:10 (Only provaccination)	Group 10:0 (Only antivaccination)	
Sex	Male	Count	4	3	1	8
		Expected Count	2.5	2.9	2.7	8.0
	Female	Count	8	11	12	31
		Expected Count	9.5	11.1	10.3	31.0
Total		Count	12	14	13	39
		Expected Count	12.0	14.0	13.0	39.0

Chi-Square Tests

	Value	df	Asymp. Sig. (2-sided)	Exact Sig. (2-sided)
Pearson Chi-Square	2.527[a]	2	.283	.294
Fisher's Exact Test	2.463			.330
N of Valid Cases	39			

a. 3 cells (50.0%) have expected count less than 5. The minimum expected count is 2.46.

ANOVA

Age

	Sum of Squares	df	Mean Square	F	Sig.
Between Groups	.805	2	.402	1.843	.173
Within Groups	7.862	36	.218		
Total	8.667	38			

Experiment 2

			Experimental Groups			
			Group 0:10 (Only provaccination)	Group 4:6	Group 6:4	Group 8:
Level of education	High School	Count	3	4	5	6
		Expected Count	5.3	8.0	5.7	5.5
	College or University	Count	27	41	26	25
		Expected Count	24.4	36.5	26.0	25.2
	Vocational Training	Count	0	0	1	0
		Expected Count	.3	.5	.3	.3
Total		Count	30	45	32	31
		Expected Count	30.0	45.0	32.0	31.0

			Experimental Groups		Total
			Group 10:0 (Only antivaccination)	Control Group (Normal Google)	
Level of education	High School	Count	7	10	35
		Expected Count	5.2	5.3	35.0
	College or University	Count	22	19	160
		Expected Count	23.6	24.4	160.0
	Vocational Training	Count	0	1	2
		Expected Count	.3	.3	2.0
Total		Count	29	30	197
		Expected Count	29.0	30.0	197.0

Chi-Square Tests

	Value	df	Asymp. Sig. (2-sided)	Exact Sig. (2-sided)
Pearson Chi-Square	14.370[a]	10	.157	.142
Fisher's Exact Test	13.714			.084
N of Valid Cases	197			

a. 6 cells (33.3%) have expected count less than 5. The minimum expected count is .29.

			Experimental Groups			
			Group 0:10 (Only provaccination)	Group 4:6	Group 6:4	Group 8:2
Have you ever had training for a job in the medical field or worked in a medical environment?	No	Count	25	36	29	25
		Expected Count	25.3	37.9	27.0	26.1
	Yes	Count	5	8	3	6
		Expected Count	4.6	6.9	4.9	4.7
	No Answer	Count	0	1	0	0
		Expected Count	.2	.2	.2	.2
Total		Count	30	45	32	31
		Expected Count	30.0	45.0	32.0	31.0

			Experimental Groups		Total
			Group 10:0 (Only antivaccination)	Control Group (Normal Google)	
Have you ever had training for a job in the medical field or worked in a medical environment?	No	Count	26	25	166
		Expected Count	24.4	25.3	166.0
	Yes	Count	3	5	30
		Expected Count	4.4	4.6	30.0
	No Answer	Count	0	0	1
		Expected Count	.1	.2	1.0
Total		Count	29	30	197
		Expected Count	29.0	30.0	197.0

Chi-Square Tests

	Value	df	Asymp. Sig. (2-sided)	Exact Sig. (2-sided)
Pearson Chi-Square	5.577[a]	10	.849	.960
Fisher's Exact Test	6.862			.909
N of Valid Cases	197			

a. 11 cells (61.1%) have expected count less than 5. The minimum expected count is .15.

			Experimental Groups			
			Group 0:10 (Only provaccination)	Group 4:6	Group 6:4	Group 8:2
Did you ever know someone who had any side effects from vaccines?	No	Count	23	34	26	24
		Expected Count	23.0	34.5	24.5	23.8
	Yes	Count	7	11	6	7
		Expected Count	6.9	10.3	7.3	7.1
	No Answer	Count	0	0	0	0
		Expected Count	.2	.2	.2	.2
Total		Count	30	45	32	31
		Expected Count	30.0	45.0	32.0	31.0

			Experimental Groups		Total
			Group 10:0 (Only antivaccination)	Control Group (Normal Google)	
Did you ever know someone who had any side effects from vaccines?	No	Count	21	23	151
		Expected Count	22.2	23.0	151.0
	Yes	Count	7	7	45
		Expected Count	6.6	6.9	45.0
	No Answer	Count	1	0	1
		Expected Count	.1	.2	1.0
Total		Count	29	30	197
		Expected Count	29.0	30.0	197.0

Chi-Square Tests

	Value	df	Asymp. Sig. (2-sided)	Exact Sig. (2-sided)
Pearson Chi-Square	6.273[a]	10	.792	.921
Fisher's Exact Test	6.056			.977
N of Valid Cases	197			

a. 6 cells (33.3%) have expected count less than 5. The minimum expected count is .15.

			Experimental Groups					
			Group 0:10 (Only provaccination)	Group 4:6	Group 6:4	Group 8:2	Group 10:0 (Only antivaccination)	Control Group (Normal Google)
Sex	Male	Count	15	22	24	20	20	20
		Expected Count	18.4	27.6	19.7	19.0	17.8	18.4
	Female	Count	15	23	8	11	9	10
		Expected Count	11.6	17.4	12.3	12.0	11.2	11.6
Total		Count	30	45	32	31	29	30
		Expected Count	30.0	45.0	32.0	31.0	29.0	30.0

			Total
Sex	Male	Count	121
		Expected Count	121.0
	Female	Count	76
		Expected Count	76.0
Total		Count	197
		Expected Count	197.0

Chi-Square Tests

	Value	df	Asymp. Sig. (2-sided)	Exact Sig. (2-sided)
Pearson Chi-Square	8.294[a]	5	.141	.142
Fisher's Exact Test	8.130			.148
N of Valid Cases	197			

a. 0 cells (0.0%) have expected count less than 5. The minimum expected count is 11.19.

ANOVA

Age

	Sum of Squares	df	Mean Square	F	Sig.
Between Groups	377.476	5	75.495	.575	.719
Within Groups	25067.377	191	131.243		
Total	25444.853	196			
